# Obstetric Risk Factors and Serological Characteristics of Early-Onset Neonates Bacterial Infections

**DOI:** 10.3389/fsurg.2022.899795

**Published:** 2022-06-20

**Authors:** Yuejiao Wang, Qi Chen, Shixia Xu, Shuang Chao

**Affiliations:** ^1^ Department of Pediatrics, Beijing Tsinghua Changgung Hospital, School of Clinical Medicine, Tsinghua University, Beijing, China; ^2^ Department of Neonatology, Shangrao Maternal and Child Healthcare Hospital of Jiangxi Province, Shangrao, China

**Keywords:** neonatal, early-onset bacterial infections, obstetrics, high-risk factors, serological features

## Abstract

**Purpose:**

To analyze the obstetric high-risk factors and serological characteristics of early-onset neonatal bacterial infections (EONBI).

**Methods:**

119 neonates with early-onset bacterial infection who were admitted to the neonatal ward of our hospital from October 2020 to December 2021 were recorded as the study group, and 100 neonates without bacterial infection who were admitted during the same period were used as the reference group. Comparative analysis of obstetric high-risk factors and serological characteristics of EONBI.

**Results:**

There was no statistical difference between the two groups in terms of gender and age at admission (*P *> 0.05). The gestational age and birth weight of newborns in the study group were lower than those in the reference group (*P *< 0.001). Comparing the maternal factors of EONBI between the two groups, there was no statistical difference in age, number of obstetric inspections, whether to use antibiotics, and mode of delivery (*P *> 0.05). Univariate analysis showed that preterm birth, unexplained asphyxia, fecal contamination of amniotic fluid, maternal infection during pregnancy, and premature rupture of membranes ≥18 h were significantly associated with EONBI (*P *< 0.05); while there was no significant difference between the two groups in the comparison between diabetic mother and child and maternal fever at delivery (*P *> 0.05). Multifactorial analysis showed that preterm birth, fecal contamination of amniotic fluid, maternal infection during pregnancy, and premature rupture of membranes ≥18 h had a good multivariate dependence on EONBI (*P *< 0.05), while there was no significant association with unexplained asphyxia, diabetic mother and child, and maternal fever at delivery (*P *> 0.05). The incidence of neonatal temperature >37.9°C was higher in the study group than in the reference group (*P *< 0.05), and there were no statistical differences in the comparison of other clinical manifestations (*P *> 0.05). The CRP level of neonates in the study group (47.33 ± 4.14) mg/L was higher than that of the reference group (4.84 ± 1.03) mg/L (*P *< 0.001). The WBC level of neonates in the study group (5.64 ± 1.18) 10^9^/L was higher than that of the reference group (0.28 ± 0.04) 10^9^/L (*P *< 0.001). The PCT level of neonates in the study group (5.41 ± 0.85) µg/L was higher than that of the reference group (0.24 ± 0.07) µg/L (*P *< 0.001).

**Conclusion:**

EONBI is closely associated with several obstetric high-risk factors, including preterm birth, fecal contamination of amniotic fluid, maternal infection during pregnancy, and premature rupture of membranes ≥18 h; EONBI has no specific symptoms and signs, but serum CRP, WBC, and PCT levels are significantly higher than those of newborns without co-infection with bacteria.

## Introduction

Newborn infants are immunocompromised and have low resistance, so bacteria can easily invade the body from the skin mucosa, umbilical cord stump, respiratory and digestive tracts causing bacterial infections in newborns, which are common types of inflammatory infections in the first 72 h of life (1, 2). Generally, the first symptom of bacterial infection in newborns is fever (3). Coughing and coughing up sputum may occur in the case of respiratory tract infections caused by bacterial infections (4). In case of bacterial infection of the intestinal tract, abdominal pain and diarrhea may be present (5). If bacterial infection such as bacteremia and sepsis occurs, it is a more serious situation, and effective antibiotic treatment is required in time.

Currently, no global assessment data on the incidence of bacterial infections in newborns are available for each country. According to incomplete statistics at home and abroad, the incidence of early-onset neonatal bacterial infections (EONBI) can range from 1% to 10% throughout live births, and once a neonate develops a systemic infection, the chance of causing its death can be 20% to 40%, and even if the child survives, 10% to 30% of the children can still be left with varying degrees of sequelae later in life (6, 7). Alerting newborns to danger signs and early identification of bacterial infections from other pathogens in a simple bedside laboratory test is of great importance to control the severity of bacterial infections in newborns and reduce neonatal mortality.

According to previous reports, in the early stages of bacterial infection, children often present with some signs, symptoms or changes in laboratory tests of inflammatory response, and these children are often accompanied by the presence of high-risk factors predisposing the newborn to bacterial infection (8). However, the clinical manifestations after infection in children are mostly not obvious, and laboratory findings are often not specific, making early diagnosis of early-onset bacterial infections in neonates relatively difficult (9). Based on the above, it is necessary to properly assess whether bacterial infections occur in the early neonatal period and to study the relationship between the occurrence of early infections and obstetric high-risk factors. This study analyzes the obstetric high-risk factors and serological characteristics of EONBI, with a view to and provides a favorable reference for its early diagnosis and timely and effective control treatment. The details are as follows.

## Materials and Methods

### Research Object

The subjects of this experiment were neonates with ≥1 of the following obstetric high-risk factors who were admitted to the neonatal ward of our hospital from October 2020 to December 2021: preterm birth, unexplained asphyxia, diabetic mother and child, fecal contamination of amniotic fluid, maternal fever at delivery (≥38°C), maternal infection during pregnancy (including third trimester vaginitis, chorioamnionitis), premature rupture of membranes ≥18 h. All children were admitted to the hospital at an age of ≤3 days and had no other obstetric high-risk factors or complications outside the scope of the above study, and no congenital anomalies.

### Grouping and Inclusion/Exclusion Criteria

The children were divided into the study group (*n* = 119) with co-infection and the reference group (*n* = 100) without co-infection according to whether they were co-infected with bacteria or not. Inclusion criteria for the study group: ① With ≥1 obstetric high-risk factor; ② At least one of the following main conditions was met: blood culture or sputum culture or peripheral secretion culture (+); one or more clinical signs of infection (e.g., the child showed signs of systemic bacterial infection such as fever, low response, reduced milk intake); ③ If the above primary condition was (−), then at least one of the following secondary conditions was satisfied: elevated C-reactive protein (CRP) >8 mg/L; increased white blood cell (WBC) >25 × 10^9^/L or decreased <5 × 10^9^/L; decreased platelets <100 × 10^9^/L. Exclusion criteria for the study group: Excluding sepsis, pneumonia, necrotizing small bowel colitis, urinary tract infection, meningitis, enteritis and impetigo and other diseases with clear etiology and site of infection, excluding infection by other pathogenic microorganisms such as viruses, mycobacteria and protozoa. Inclusion criteria for the reference group: children with obstetric high-risk factors but no clinical manifestations of infection, negative laboratory indicators, and clinical exclusion of infection.

### Observation Items

Baseline information was collected upon admission of the child, including gender, age at admission, gestational age, and birth weight. Maternal factors that might be present in EONBI were collected, including age, number of obstetric inspections, whether to use antibiotics, and mode of delivery, etc. The obstetric high-risk factors present in the two groups of newborns were counted, and the relationship between EONBI and obstetric high-risk factors was analyzed by univariate and multiple logistic regression. Both groups of neonates were observed for clinical manifestations and examined for CRP, blood routine and blood culture before the administration of antibiotics. If there were clinical manifestations, sputum culture, umbilical or ocular secretion culture or chest X-ray were added according to the condition of the child. Comparison of CRP, WBC, and calcitoninogen (PCT) levels prior to antibiotic administration in both groups to assess their serological characteristics. The assay was performed by collecting 5 mL of fasting elbow vein blood, centrifuging it at 2,000 r/min for 10 min, and finally storing the separated plasma in a refrigerator at −20°C. 7,600 Hitachi automatic biochemical analyzer (Beijing Tailin Oriental Trading Co., Ltd.) and immunoturbidimetry were used to detect CRP and PCT levels, AU5800 automatic blood analyzer [Beckman Coulter Trading (China) Co., Ltd.] and supporting reagents were used to detect WBC levels.

### Treatment Methods

The neonates in both groups were monitored continuously for 72 h, and they were given antibiotics of the third generation or more of cephalosporin immediately after infection for 1–3 weeks. Those who ruled out infection were given lower antibiotics or no antibiotics, paying attention to intensive care of local infection.

### Statistical Methods

SPSS 22.0 software was applied for statistical analysis, and the measurement data were expressed as mean ± standard deviation, and paired t-test and ANOVA were performed. The statistical data were expressed as ratios, and the *χ*^2^ test was performed. In order to exclude the influence of confounding factors on the results of the study, with EONBI as the dependent variable, the relationship between EONBI and 7 obstetric high-risk factors was analyzed by multivariate Logistic regression. *P *< 0.05 was considered statistically significant.

## Results

### Comparison of Baseline Information Between the Two Groups of Newborns

**Figure 1:**
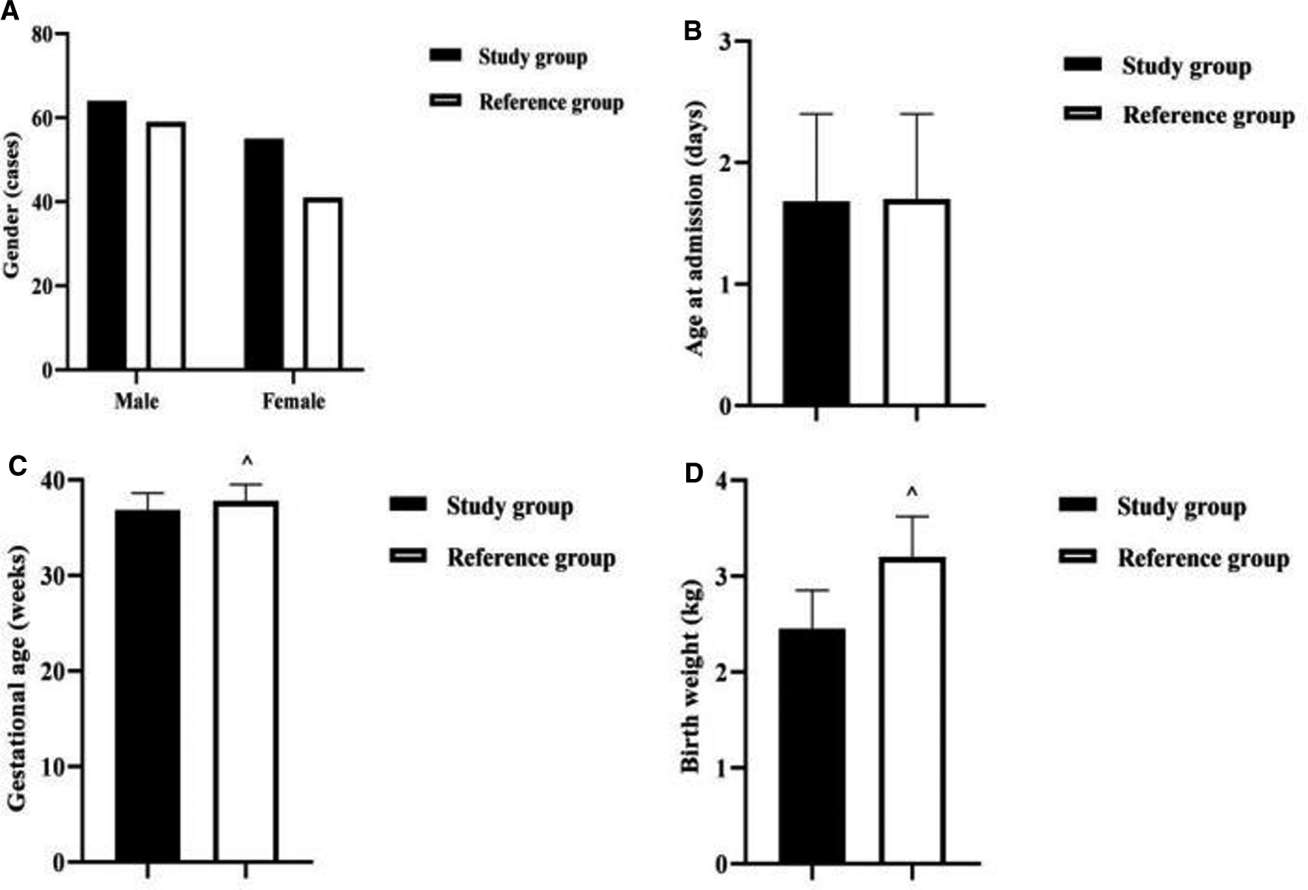
Comparison of baseline information between the two groups of newborns. Note: (**A**) Comparison of neonatal gender between the two groups. (**B**) Comparison of neonatal age at admission between the two groups. (**C**) Comparison of neonatal gestational age between the two groups. (**D**) Comparison of neonatal birth weight between the two groups. Comparison with the study group ^^^*P *< 0.001.

There was no statistical difference between the two groups in terms of gender and age at admission (*P *> 0.05). The gestational age and birth weight of newborns in the study group were lower than those in the reference group (*P *< 0.001) (Figure 1).

### Analysis of the Maternal Factors of EONBI

**Figure 2:**
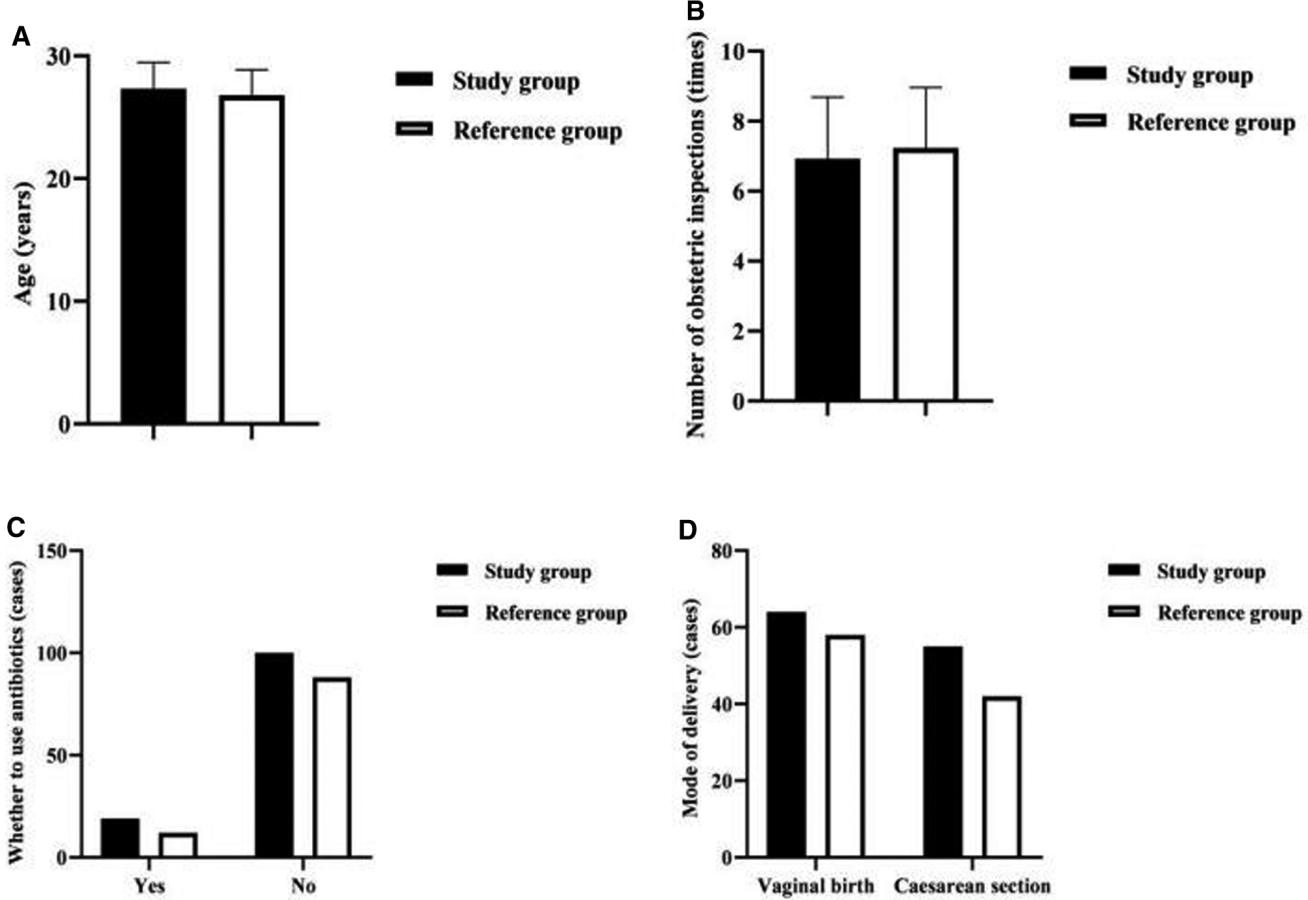
Analysis of the maternal factors of EONBI. Note: (**A**) Comparison of maternal age in both groups (years). (**B**) Comparison of the number of obstetric inspections in both groups (times). (**C**) Comparison of whether to use antibiotics in both groups (cases). (**D**) Comparison of the mode of delivery in both groups (cases).

Comparing the maternal factors of EONBI between the two groups, there was no statistical difference in age, number of obstetric inspections, whether to use antibiotics, and mode of delivery (*P *> 0.05) (Figure 2).

### Univariate Analysis of EONBI and Obstetric High-Risk Factors

Univariate analysis showed that preterm birth, unexplained asphyxia, fecal contamination of amniotic fluid, maternal infection during pregnancy, and premature rupture of membranes ≥18 h were significantly associated with EONBI (*P *< 0.05); while there was no significant difference between the two groups in the comparison between diabetic mother and child and maternal fever at delivery (*P *> 0.05) (Table 1).

**Table 1:** Univariate analysis of EONBI and obstetric high-risk factors.

High-risk factors	Study group (*n* = 119)		Reference group (*n* = 100)		*χ* ^2^	*P*
	*n*	%	*n*	%		
Preterm birth	85	71.43	31	31.00	35.651	<0.001
Unexplained asphyxia	71	59.66	15	15.00	45.452	<0.001
Diabetic mother and child	19	15.97	11	11.00	1.134	0.287
Fecal contamination of amniotic fluid	86	72.27	35	35.00	30.526	<0.001
Maternal fever at delivery	33	27.73	35	35.00	1.341	0.247
Maternal infection during pregnancy	42	35.29	11	11.00	17.483	<0.001
Premature rupture of membranes r18 h	104	87.39	43	43.00	48.530	<0.001

### Multifactorial Analysis of EONBI and Obstetric High-Risk Factors

Multifactorial analysis showed that preterm birth, fecal contamination of amniotic fluid, maternal infection during pregnancy, and premature rupture of membranes ≥18 h had a good multivariate dependence on EONBI (*P *< 0.05), while there was no significant association with unexplained asphyxia, diabetic mother and child, and maternal fever at delivery (*P *> 0.05) (Table 2).

**Table 2:** Multifactorial analysis of EONBI and obstetric high-risk factors.

High-risk factors	*β*	SE	Wald	*P*	OR	95% CI	
						Lower limit	Upper limit
Preterm birth	0.247	0.036	6.309	0.012	1.280	1.193	1.374
Unexplained asphyxia	1.450	0.770	3.681	0.055	4.263	0.943	19.283
Diabetic mother and child	1.745	1.253	0.281	0.596	5.726	0.491	66.745
Fecal contamination of amniotic fluid	3.141	1.385	11.449	0.001	23.127	1.532	349.184
Maternal fever at delivery	1.823	1.788	0.137	0.712	6.190	0.186	205.918
Maternal infection during pregnancy	4.265	1.023	19.625	<0.001	71.165	9.582	528.520
Premature rupture of membranes ≥18 h	2.520	1.122	7.864	0.005	12.429	1.378	112.070

### Analysis of Clinical Manifestations in Two Groups of Newborns

The incidence of neonatal temperature >37.9°C was higher in the study group than in the reference group (*P *< 0.05), and there were no statistical differences in the comparison of other clinical manifestations (*P *> 0.05) (Table 3).

**Table 3:** Analysis of clinical manifestations in two groups of newborns.

Clinical manifestations	Study group (*n* = 119)		Reference group (*n* = 100)		*χ* ^2^	*P*
	*n*	%	*n*	%		
Body temperature <36°C	5	4.20	3	3.00	0.223	0.637
Body temperature >37.9°C	18	15.13	4	4.00	7.444	0.006
Breathing >60 breaths/min	1	0.84	1	1.00	0.015	0.902
Heart rate <100 or >160 Beats/min	10	8.40	9	9.00	0.024	0.876
Primitive reflections Weaken or disappear	1	0.84	2	2.00	0.541	0.462
Poor feeding	15	12.61	8	8.00	1.226	0.268
Breast rejection	2	1.68	1	1.00	0.186	0.666
Bloating	1	0.84	1	1.00	0.015	0.902
Vomiting	7	5.88	9	9.00	0.780	0.377
Breathing difficulties	1	0.84	1	1.00	0.015	0.902
Poor response	10	8.40	6	6.00	0.464	0.496
No or less crying	3	2.52	1	1.00	0.701	0.402
Irritable and irritable	7	5.88	4	4.00	0.404	0.525
Pale gray face	8	6.72	9	9.00	0.394	0.530

### Analysis of Serological Characteristics of Two Groups of Newborns

**Figure 3:**
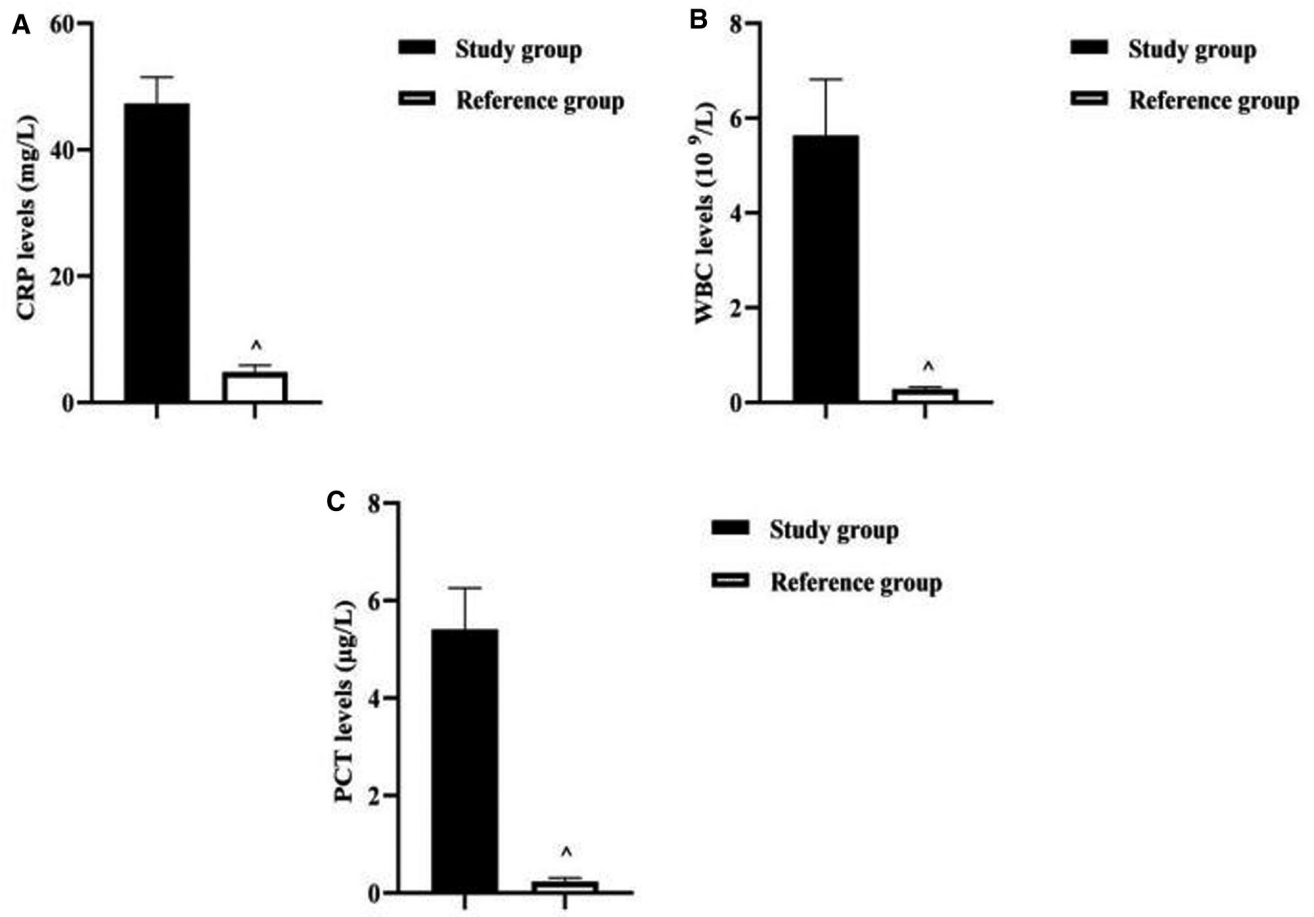
Analysis of serological characteristics of two groups of newborns. Note: (**A**) Comparison of CRP levels between two groups of neonates. (**B**) Comparison of WBC levels between two groups of neonates. (**C**) Comparison of PCT levels between two groups of neonates. Comparison with the study group ^*P *< 0.001.

The CRP level of neonates in the study group (47.33 ± 4.14) mg/L was higher than that of the reference group (4.84 ± 1.03) mg/L (*P *< 0.001). The WBC level of neonates in the study group (5.64 ± 1.18) 10^9^/L was higher than that of the reference group (0.28 ± 0.04) 10^9^/L (*P *< 0.001). The PCT level of neonates in the study group (5.41 ± 0.85) µg/L was higher than that of the reference group (0.24 ± 0.07) µg/L (*P *< 0.001) (Figure 3).

## Discussion

The clinical symptoms of early neonatal bacterial infections are atypical. At present, the diagnostic criteria for neonatal bacterial infection at home and abroad are mostly based on the clinical manifestations of severe infection or even sepsis. There is a lack of scientificity and consistency in the early judgment of infection, and there are often two drawbacks: One is the failure to detect bacterial infections in newborns in a timely manner, leading to the spread of infection; the other is the use of antibiotic treatment for all newborns with obstetric high-risk factors, leading to the misuse of antibiotics (10). As seen above, it is extremely important to clarify the obstetric high-risk factors of EONBI and its infection status for the smooth implementation of the next treatment.

The results of this paper showed that preterm birth, unexplained asphyxia, fecal contamination of amniotic fluid, maternal infection during pregnancy, and premature rupture of membranes ≥18 h were significantly associated with EONBI. Preterm delivery has been reported as a high risk factor significantly associated with EONBI and 64% of fecal contamination of amniotic fluid is associated with chorioamnionitis (11). The present study confirmed this finding, analyzing the reasons for this, premature infants have immature immune systems and all systems, and are more likely to acquire infections via the above-mentioned routes or through the skin and respiratory tract. In neonatal asphyxia, humoral and cellular immune functions are impaired, and the number of invasive operations during treatment predisposes to bacterial invasion, resulting in early bacterial infections (12). And EONBI mostly originates from intrauterine and is closely related to fecal contamination of amniotic fluid, maternal infection during pregnancy, premature rupture of membranes, etc. The infection routes include maternal hematogenous spread, ascending infection, retrograde fallopian tube implantation, invasive operation, etc. (13, 14). The most common of these are episodic infections, such as those caused by the upward movement of bacteria in the birth canal after premature rupture of membranes, and when bacteria spread to the amniotic cavity and grow in the amniotic fluid, the fetus can also become ill by inhaling contaminated amniotic fluid before and during delivery (15, 16).

The multifactorial analysis in this paper shows that preterm birth, fecal contamination of amniotic fluid, maternal infection during pregnancy, and premature rupture of membranes ≥18 h had a good multivariate dependence on EONBI, and the most significant risk factor for EONBI is maternal infection during pregnancy. This further confirms that genital tract infection during maternal pregnancy is an important factor in triggering intrauterine infection (17). During intrauterine infection, bacteria and their products stimulate the mRNA expression of cytokines such as IL-1β, IL-6, IL-8, and TNF-α in the amniotic membrane and trophoblast cells, which induce preterm labor and lead to EONBI (18). In this study, the OR value of maternal infection during pregnancy was 71.165, which suggested that the risk of EONBI in the presence of this high-risk factor was 71.165 times higher than that in the absence of this high-risk factor. Unexplained asphyxia was not significantly associated with EONBI after controlling for confounders. This may be because the rapid development of aseptic technique and medical devices in recent years has led to a decrease in infections acquired through resuscitation.

In recent years, there has been a significant increase in the prevalence of gestational diabetes, which has been reported to be as high as 5.8% to 25.1% (19). As the placenta is rich in blood supply, the number of villi increases, the villi gap narrows accordingly, and cells proliferate, making the circulatory barrier between mother and fetus widen and the small metaplastic arteries narrow, while the potential microangiopathy in pregnant women with diabetes can aggravate the obstruction of small arteries on the fetal side of the villi stem, leading to chronic intrauterine hypoxia, acidosis, and secondary infection in the fetus (20). However, the present results after controlling for confounders showed no significant association between diabetic mother and child and EONBI. This may be related to the popularization of gestational diabetes screening in my country and the vigorous intervention of gestational diabetes in recent years. The present results also showed that maternal fever at delivery was also not significantly associated with EONBI. This may be related to the higher priority given to it by obstetricians and therefore earlier intervention. It is also possible that the sample size in this study was limited and further studies with larger sample sizes are needed to understand more precisely the relationship between the two. In this result, the gestational age and birth weight of newborns in the study group were lower than those in the reference group. This may be due to the fact that 71.43% of the newborns in the study group were preterm, much higher than the 31.00% in the reference group, so the children were under gestational age and most of them had a birth weight of no more than 2.5 kg. In this paper, the incidence of neonatal temperature >37.9°C was higher in the study group than in the reference group, and there were no statistical differences in the comparison of other clinical manifestations. This further confirms that the clinical signs and symptoms of EONBI are atypical and that the first symptom of bacterial infection in newborns is fever.

Previously, the clinical diagnosis of neonatal infectious diseases was based on the detection of peripheral blood WBC counts, and the results of WBC detection were affected by various factors, and some neonates had poor WBC count bases, and the detected WBC levels were still in the normal range even though bacterial infections had occurred in the body, so WBC could only be used as a routine reference index for the diagnosis of infectious diseases (21, 22). CRP, synthesized by inflammatory factors stimulating hepatocytes, is an acute phase response protein that triggers immunomodulation, phagocytosis, formation of immune complexes, and activation of the complement system, and has been clinically used as a nonspecific marker of the systemic inflammatory response (23). When inflammatory reaction or tissue damage occurs in the organism, CRP levels can rise abruptly within hours or 1–2 days, and its level is positively correlated with the degree of infection in the organism, so it is often used as an early diagnostic indicator of bacterial infection (24). Besides, in recent years, PCT has been considered as an ideal serological marker for the diagnosis of bacteriological infections, which is considered to have not only high specificity and sensitivity, but also can compensate for the lack of lag in bacterial culture, thus facilitating the accurate and rapid diagnosis and treatment of diseases (25). The levels of CRP, WBC, and PCT in the newborns of this result study group were higher than those of the reference group. This suggests that health care providers can use this serological characteristics as an important reference indicator to identify the presence of bacterial infection in newborns.

## Conclusion

EONBI is closely associated with several obstetric high-risk factors, including preterm birth, fecal contamination of amniotic fluid, maternal infection during pregnancy, and premature rupture of membranes ≥18 h; EONBI has no specific symptoms and signs, but serum CRP, WBC, and PCT levels are significantly higher than those of newborns without co-infection with bacteria.

## Data Availability

The original contributions presented in the study are included in the article/Supplementary Material, further inquiries can be directed to the corresponding author/s.
